# A Fast Alternative to Soft Lithography for the Fabrication of Organ‐on‐a‐Chip Elastomeric‐Based Devices and Microactuators

**DOI:** 10.1002/advs.202003273

**Published:** 2021-02-08

**Authors:** Daniel A. Ferreira, Mario Rothbauer, João P. Conde, Peter Ertl, Carla Oliveira, Pedro L. Granja

**Affiliations:** ^1^ i3S – Instituto de Investigação e Inovação em Saúde Universidade do Porto Rua Alfredo Allen, 208 Porto 4200‐135 Portugal; ^2^ INEB – Instituto de Engenharia Biomédica Universidade do Porto Rua Alfredo Allen, 208 Porto 4200‐135 Portugal; ^3^ ICBAS – Instituto de Ciências Biomédicas Abel Salazar Universidade do Porto Rua Jorge de Viterbo Ferreira, 228 Porto 4050‐313 Portugal; ^4^ Department of Orthopedics and Trauma Surgery Karl Chiari Lab for Orthopedic Biology Medical University of Vienna Währinger Gürtel, 18‐20 Vienna 1090 Austria; ^5^ Institute of Applied Synthetic Chemistry Vienna University of Technology (TUW) Getreidmarkt, 9/163 Vienna 1060 Austria; ^6^ Department of Bioengineering Instituto Superior Técnico Universidade de Lisboa Av. Rovisco Pais, 1 Lisboa 1049‐001 Portugal; ^7^ Instituto de Engenharia de Sistemas e Computadores – Microsistemas e Nanotecnologia (INESC MN) Rua Alves Redol, 9 Lisboa 1000‐029 Portugal; ^8^ Faculty of Technical Chemistry Vienna University of Technology (TUW) Getreidemarkt 9 Vienna 1060 Austria; ^9^ Ipatimup – Institute of Molecular Pathology and Immunology Universidade do Porto Rua Júlio Amaral de Carvalho 45 Porto 4200‐135 Portugal; ^10^ Department of Pathology Faculty of Medicine University of Porto Alameda Prof. Hernâni Monteiro Porto 4200‐319 Portugal

**Keywords:** fast prototyping, microactuators, microfluidics, organ‐on‐a‐chip, pdms

## Abstract

Organ‐on‐a‐chip technology promises to revolutionize how pre‐clinical human trials are conducted. Engineering an in vitro environment that mimics the functionality and architecture of human physiology is essential toward building better platforms for drug development and personalized medicine. However, the complex nature of these devices requires specialized, time consuming, and expensive fabrication methodologies. Alternatives that reduce design‐to‐prototype time are needed, in order to fulfill the potential of these devices. Here, a streamlined approach is proposed for the fabrication of organ‐on‐a‐chip devices with incorporated microactuators, by using an adaptation of xurography. This method can generate multilayered, membrane‐integrated biochips in a matter of hours, using low‐cost benchtop equipment. These devices are capable of withstanding considerable pressure without delamination. Furthermore, this method is suitable for the integration of flexible membranes, required for organ‐on‐a‐chip applications, such as mechanical actuation or the establishment of biological barrier function. The devices are compatible with cell culture applications and present no cytotoxic effects or observable alterations on cellular homeostasis. This fabrication method can rapidly generate organ‐on‐a‐chip prototypes for a fraction of cost and time, in comparison to conventional soft lithography, constituting an interesting alternative to the current fabrication methods.

## Graphical abstract

A microfabrication method based on xurography, capable of producing complex multilayered devices with integrated microactuators, suitable for organ‐on‐a‐chip applications, in a matter of hours is described. Microdevices fabricated with the technology are biocompatible and capable of mechanical actuation at physiological levels of strain. The method provides a fast alternative to current soft lithography methods, by reducing fabrication cost and time.

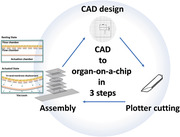

## 1 Introduction

Microfluidic devices are well established as experimental platforms in life sciences. Devices designed for cell sorting, DNA sequencing, electrophoresis, 3D cell culture are just a few examples of what can be achieved at the microscale.^[^ ^1^, ^2^ ^]^ Miniaturization of biological procedures has many advantages. Not only does it reduce the bench footprint, it also requires minute quantities of reagents, solvents and biological samples. Moreover, these systems provide the ability to control aspects of the cell microenvironment at more relevant kinetic and spatial scales.^[^ ^3^ ^]^ The design versatility and scalability of microfluidic platforms, through parallelization of devices in array‐like designs,^[^ ^4^, ^5^, ^6^ ^]^ make them a powerful tool in any life sciences laboratory.

Recently, the concept of organ‐on‐a‐chip devices was introduced.^[^ ^7^ ^]^ These microfluidic systems are far more complex than cell‐based lab‐on‐a‐chip devices, as they aim to recreate the complex micro‐physiological architecture and function of the organ they intend to emulate.^[^ ^8^, ^9^, ^10^ ^]^ Importantly, the successful integration of microactuators within organ‐on‐a‐chip devices, allowed the application of well‐defined and cyclical strain on the cell culture substrate. The ability to control the intensity, duration and pattern of the mechanical forces within the system, make organ‐on‐a‐chip platforms, a powerful tool to understand how mechanical transduction affects cellular response at the tissue level, thus modulating to a greater extent a key aspect of the in vivo native microenvironment, i.e., the cellular response to biomechanical cues. Assessing the impact of mechanical forces at the cellular level and understanding how cells transduce these mechanical forces into biochemical signals, in a physiologically relevant context, is in fact one of the most innovative aspects of the organ on a chip technology, and of particular interest when emulating the in vivo microenvironment of tissues exposed to strain.^[^ ^11^, ^12^ ^]^ The relevance of mechanotransduction has been highlighted in several studies where microfluidic platforms were developed to simulate physiological levels of strain.^[^ ^7^, ^13^, ^14^, ^15^ ^]^ Despite the obvious advantages of the technology, design of organ‐on‐a‐chip microdevices is a complex procedure. Their three‐dimensional (3D) layout and incorporation of several tissue‐like features, such as a simplified stromal component and epithelial barrier architecture, is further complicated by the incorporation of embedded mechanical microactuators.^[^ ^7^, ^11^, ^13^, ^14^ ^]^ Creating complex structures that correctly emulate the biological counterparts, usually implies fabricating multilayered devices, with two or more chambers separated by porous membranes, as well as complex flexible mechanisms that serve as mechanical actuators. Also, replicating some organ functions requires the design of intricate microchannel geometries to house, in a specific layout, the individual components of the organotypic unit being emulated^[^ ^16^, ^17^ ^]^ or to manipulate diffusion distances.^[^ ^5^, ^18^ ^]^ While geometry design, by itself, is not constrained by fabrication limitations, it is essential that they are amenable to correct cell culture maintenance and homeostasis.^[^ ^19^ ^]^ Methods with a fast turnaround time from design to device, are key to reduce experimental costs at the early stages of organ‐on‐a‐chip design. The challenge remains to develop a technology that allows the fast generation of workable prototypes, while retaining the characteristics of devices produced by soft lithography. Notably, the optical transparency of cured polydimethylsiloxane (PDMS), as well as its soft elastomeric nature, that is a key feature for organ‐on‐a‐chip devices with embedded mechanical actuation.

The present study establishes an innovative method to fabricate complex multilayered PDMS fluidic devices with integrated microactuators, suitable for organ‐on‐a‐chip applications. The technique used is based on xurography^[^ ^20^ ^]^ and relies in the machining of PDMS laminates using a benchtop cutting plotter as previously described.^[^ ^21^ ^]^ The approach simplifies the entire fabrication procedure into 3 steps: design, machining and assembly. The devices produced with this method were characterized regarding the performance and biocompatibility of the materials and procedures, in view to establish a fast and efficient fabrication method for organ‐on‐a‐chip applications. To this end, we thoroughly characterized the fabrication process regarding machining resolution, resistance to delamination and ability of these devices to sustain a long term epithelial gastric cell line (MKN74) culture. This was followed by the integration of microactuators within the chip and characterization of their reliability in reproducing biomechanical cues at physiological levels, as well as their ability to operate as in‐line degassers to eliminate on‐chip air bubbles.

The proposed method generates fully operational biochips, capable of sustaining long‐term operation under cell culture conditions. Furthermore, they can be adapted to complex organ‐on‐a‐chip designs, as shown by the successful integration of intercalating porous membranes, mechanical actuators and in‐line degassers.

## 2 Results and Discussion

### 2.1 Design and Fabrication of a Cost‐ and Time‐Effective Multilayered Organ‐on‐a‐Chip Devices with Built‐In Microactuators

**Figure 1:**
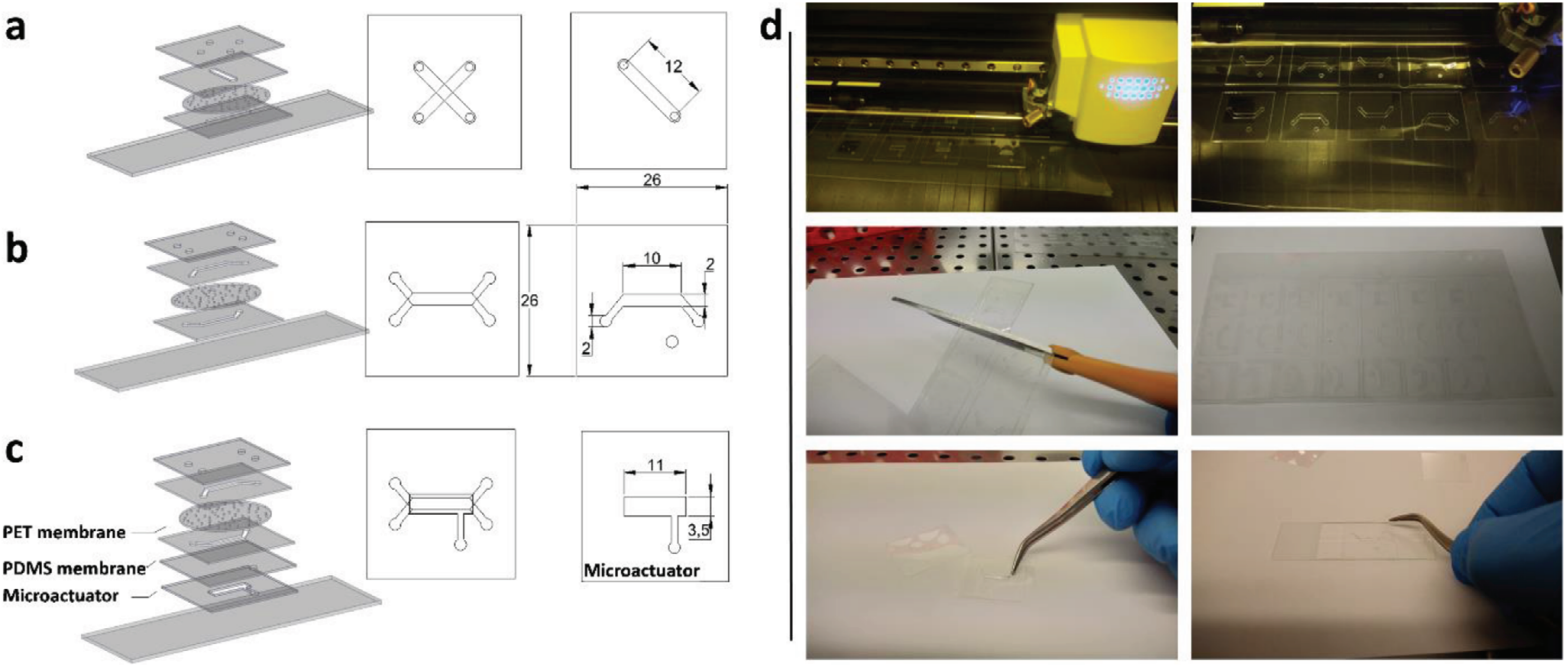
Exploded 3D view of a) Device 1, b) Device 2, and c) Device 3, each depicted with a top‐down view of the assembled structure to the right and corresponding measurements for the main fluidic and microactuator features. (values in millimeters). d) Depiction of the fabrication method. Upon completion of the chip design using CAD software, a PDMS laminated sheet is fed into a cutting plotter. After the plotting process, each sheet holds several copies of a single chip, with each squared section corresponding to a level of the chip. Each layer is then manually cut from the laminated sheet and the offcuts are removed to reveal the microfluidic channels. The chip is then carefully mounted on top of a glass microscope slide.

In this study, we developed a fast and inexpensive method to fabricate complex multilayered devices incorporating microactuators. Our approach is based on xurography, a technique that consists on the removal of material from thin film plates using a cutting plotter. Xurography was first developed as a technique to produce soft lithography molds.^[^ ^20^, ^22^ ^]^ Given its versatility and speed of use, the technique was quickly adapted to directly fabricate chip elements.^[^ ^21^, ^23^, ^24^ ^]^ Here, we used such an approach, by using a cutting plotter to directly machine thin, pre‐cured laminated PDMS sheets. Each piece cut corresponds to one layer of the chip (**Figure** **1**a–c). A single plotter run, over a 9×20 cm area of PDMS laminate, can produce as much as 7 units of a double channel prototype. Machining time varies with the complexity of the structure being cut and the cutting route the blade performs, as determined by the user. Within a few seconds, all the PDMS layers required for a complete device can be produced. This obviates the most time‐consuming step of soft lithography, namely the procedure of mold making and the casting and curing of the structural pieces, a process that, given the complexity of the tasks, can take up to two days for a double layered chip. Device assembly can then be performed following conventional soft lithography techniques, without any additional fabrication steps. Similarly to the soft lithographic process, bonding of the individual parts is dependent on the materials used, thus curing times will vary accordingly. The complete fabrication procedure is condensed in 3 steps: design, cutting and assembly (Figure 1d). Timewise, this is a major improvement on current fabrication methods of complex organ‐on‐a‐chip devices with incorporated microactuators. Recent publications have also shown promising results regarding a novel adaptation of xurography, by using biocompatible adhesive tape as the building materials.^[^ ^23^, ^24^ ^]^ Structures cut from adhesive tape, can be readily bonded, without requiring chemical treatment of the surface. Plotting of structures and complete chip assembly can be accomplished within minutes. Stallcop and colleagues,^[^ ^23^ ^]^ have also demonstrated applicability of the technique for the fabrication of simple double layered open devices with successful integration of membranes, similar to a transwell configuration. From our experience however, although faster, the technique is not suited for the fabrication of microactuators. We found that PDMS membranes used as micro‐diaphragms, bond well to adhesive tapes, but do not withstand continuous cyclical actuation (data not shown).

### 2.2 Rapid Prototyping by Plotting Allows Adequate Replication Resolution of Micropatterning

**Figure 2:**
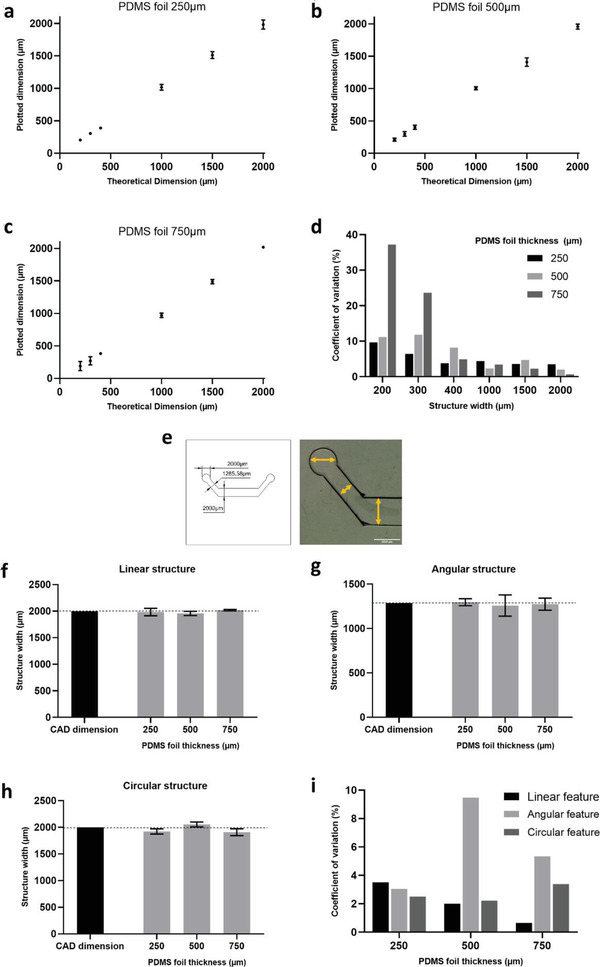
Assessment of resolution limits of the xurographic process. a–c) CAD theoretical dimension was compared to plotted dimensions across a series of rectangular structures with defined size, cut from PDMS foil sheets of 250, 500, and 750 µm thick. Results are plotted as average dimension ± SD (*n* = 4). Error bars not shown when the SD is smaller than the graphical symbol. d) Variation to CAD size is represented as the coefficient of variation (%), for the conditions detailed in (a–c), as a function of structure width. e) CAD design with theoretical dimensions and stereomicroscope image of a plotted structure. f–h) Influence of geometry on resolution assessed by measuring linear, angular and circular features as shown on e) and compared to theoretical CAD size (left most bar for each graph). Results were presented as average dimension ± SD (*n* = 4). i) Deviation to CAD size is represented as the coefficient of variation (%), for the conditions detailed in (f–h), as a function of PDMS sheet height.

One important aspect to benchmark is the ability of this technology to reproduce with precision the computer aided design (CAD) projected features, namely the original design sizing. This is required to ensure that the predicted pressure and shear stress are as close as possible to the simulated conditions. Here we characterized the lower limit of resolution by cutting in 250, 500 and 750 µm thick PDMS sheets, a series of rectangular shapes, ranging in width from 2000 µm, corresponding to original chip scaling, down to 200 µm (*n* = 4). CAD theoretical dimensions were compared to real plotted dimensions across the range for all 3 PDMS thicknesses (**Figure** **2**a–c). A width of 200 µm was the lowest achievable spacing with precision, below which, the blade starts rupturing the structure. This is similar to the results previously reported by Cosson and colleagues, for similar structures cut from thin PDMS sheets.^[^ ^21^ ^]^ We observed lower variation to CAD design for larger structures, with deviations of 3.5, 2.0 and 0.6% for 250, 500, 750 µm thicknesses respectively (Figure 2d). Deviation to CAD theoretical dimension is conserved below 8% for 400 µm wide structures, on all thicknesses tested. Bellow 400 µm, cut accuracy starts to deteriorate, in particular for thicker PDMS sheets. Our results demonstrate that smaller structures, less than 400 µm wide can be reliably obtained using PDMS sheets with a thickness of 500 µm or lower (Figure 2d). To further characterize the method, we analyzed the impact of geometry on resolution. Using our chip design (Figure 2e), we measured the average size of the 3 most common geometric shapes, cut from 250, 500 and 750 µm thick PDMS sheets. We compared the width of linear and angular structures and the diameter of circular features, with the theoretical size from the CAD generated designs, namely 2000.00 µm for linear and circular features and 1285.58 µm for the angular access channels (Figure 2e‐h). Deviation to CAD size was higher for angular structures, in particular for thicker substrates, namely 500 µm or higher. Nevertheless, variation was kept within acceptable levels, with a maximum of 9.5% variation for angular structures on 500 µm thick PDMS sheet (Figure 2i). The plotter copes better with linear and circular structures. For both geometries, variation was below 4%, irrespective of the thickness of the PDMS substrate, with the highest variation to CAD size registered for linear features cut from 250 µm thick PDMS sheets at 3.5% (Figure 2i).

Overall, our results show that precision of the machined structures cannot match that of photolithography and soft lithography, where the minimum feature size achievable ranges around 100 nm and lower.^[^ ^25^ ^]^ The stronger point of the xurographic process resides rather in its low cost, speed of fabrication and flexibility of the process, as designs can be changed and tested quickly. Xurography limits are within the micrometric range which is nevertheless, well suited for cell culture based microfluidic applications. Structures of ca. 200 µm can be effectively cut, although for better resolution, sizes of 400 µm or higher should be used, especially when using thicker PDMS sheets. We did not observe major constrains to resolution regarding the geometries tested. For larger structures, between 1285 and 2000 µm, resolution was shown to vary within acceptable intervals, regardless of geometry or the thickness of the PDMS substrate.

### 2.3 Optimal Layer Bonding is Achieved by a Combination of O_2_ Plasma Exposure and Silanization

**Figure 3:**
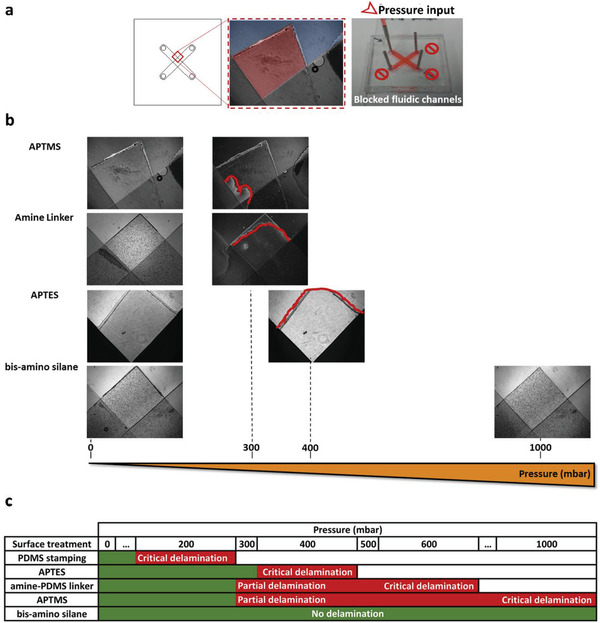
Pressure test for silanization bonding of PET membranes against PDMS laminates a) Device 1 drawing, used to assess internal pressure resistance. The red square represents the photographed area to document the experiment (left). Magnification of the monitored zone, showing PDMS‐PDMS contacts highlighted in blue and PET‐PDMS contacts outlined in red (center). Experimental layout with chip filled with dyed double distilled water. Three of the 4 outlets were blocked, and the system connected to a pressure generator (right). b) Assessment of delamination over an increasing pressure range for the 4 silanes tested. Red outline depicts zones of delamination. c) Table summarizing results of the delamination tests. Critical delamination was defined as complete detachment of the PET‐PDMS contact area, while partial delamination was defined as only partial detachment of that contact region.

Devices entirely composed of laminated PET/PDMS plates require optimal bonding between all the layers. This promotes undisturbed fluid flow and ensures that cells are contained within their designated culture chamber, without unintended extravasation to the surrounding areas. Here, we have assessed the adhesion strength of both the PDMS‐PDMS and the PET‐PDMS bonds. For this purpose, we used Device 1, a two level, cross shaped PDMS device, with a PET membrane sandwiched between the two perfusion channels as described above (**Figure** **3**a). Bonding efficacy at room temperature and normal atmosphere was tested against a pressure range between 0 and 1000 mbar, in incremental 100 mbar steps. PDMS‐PDMS bonding was performed by oxygen plasma oxidation.^[^ ^26^ ^]^ The treatment replaces the methyl chemistry at the surface of polymerized PDMS with OH groups, thus establishing Si‐OH bonds. These groups react with similar groups on the opposing plasma exposed surface, to form covalent Si‐O‐Si bonds, constituting a watertight, irreversible seal between two PDMS plates. The oxygen plasma treated surfaces, performed very well at standard operation pressure (between 10 and 50 mbar). No observable delamination on PDMS‐PDMS contacts was observable, even at a high pressure of 300 mbar. Furthermore, pressure was ramped up to 1000 mbar, the maximum pressure achieved by the pressure controller in use, without delamination (Figure 3b—refer to blue overlay region on Figure 3a to identify PDMS‐PDMS contacts).

The PET‐PDMS bond is a potentially critical weak spot in the structural integrity of the chip, as bonding two dissimilar materials may result in a weak or partial bond. Plasma oxidation proved to be insufficient for PET‐PDMS adhesion at basal cell culture working conditions, with delamination observed early on during the chip operation. Therefore, we evaluated the effectiveness of PDMS stamping and silane coupling as alternative methods to establish a pressure, temperature and humidity resistant PET‐PDMS bond. PDMS stamping uses uncured PDMS as a mortar layer applied between PDMS plates, in order to promote the embedding of the plastic material within the PDMS polymeric structure.^[^ ^27^ ^]^ This technique has the advantage of not requiring any chemical treatment of the PET membrane. Our results show that PDMS stamping produced a pressure resistant bond, as we were able to operate chips up to 200 mbar without delamination (Figure 3c). However, we observed that the PDMS‐PDMS contact is compromised with the application of PDMS stamping. The PDMS mortar does not self‐level during the stamping procedure and the PDMS mortar, when cured creates a rise on the surface. Furthermore, the PDMS mortar occasionally provokes the occlusion of the porous PET membrane or the fluidic channel, yielding a low success rate during fabrication. Therefore, we did not further test this technique. Silane coupling is described as an effective method to permanently bond plastic membranes to PDMS. This method is compatible with oxygen plasma activation and well suited to bond polymers whose surface can be readily hydroxylated, such as PET membranes.^[^ ^28^, ^29^ ^]^ Here, we tested a variety of trialkoxysilanes (APTMS and APTES),^[^ ^28^, ^30^ ^]^ a dipodal silane (bis‐amino silane) ^[^ ^31^ ^]^ and a linker incorporating an amine functionality at one terminal and a low molecular weight PDMS on the opposite terminal (amine‐PDMS linker).^[^ ^32^ ^]^ These silanes promote a chemical bond between PET and PDMS and were assayed regarding their ability to produce a stable bond under pressure and during cell culture operation. APTMS and APTES and bis‐amino silane establish C‐Si‐O bonds with the oxygen activated PET surface, thus creating a chemistry that can be further hydroxylated to establish a Si‐O‐Si bond, thereby permanently binding the PET and PDMS surfaces. A notable particularity of the bis‐amino silane, is the presence of 6 alkoxysilane reactive groups per silane molecule, resulting in the increase of its adhesive capacity. The amine‐PDMS linker works as a single step reaction, not requiring previous hydroxylation of the plastic surface. Amine‐PDMS linker forms an urethane linkage with the plastic surface, exposing a reactive PDMS group that can be bound directly to an opposite O_2_ plasma‐exposed PDMS surface. Our results indicate that surface amino functionalization with amine‐PDMS linker, APTMS and bis‐amino silane generated the strongest PET‐PDMS bonds. These 3 silanes were the top performers, withstanding pressures up to 400 mbar with only partial or no delamination of the bond. In addition, bis‐amino silane performed consistently and without delamination even at 1000 mbar (Figure 3b—refer to red overlay region on Figure 3a to identify PET‐PDMS contacts). Figure 3c summarizes the conditions tested and results obtained for each of them.

**Figure 4:**
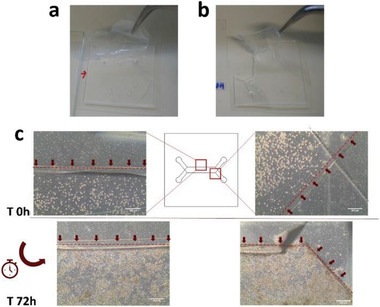
Silanization test under cell culture conditions. a) Membrane silanized with APTMS, as compared to b) a membrane silanized with bis‐amino silane. The latter depicting full adhesion between both materials, with the PDMS tearing when the PET membrane is forcefully pulled. c) Bond adhesiveness assessment at time of cell seeding (T0h) and T72h post‐seeding with no observable cell extravasation. Red arrows indicate the outline of the cell culture chamber.

To test the ability of the PET‐PDMS bond to perform well at physiological conditions, which is essential for organ‐on‐a‐chip operation, we assayed the bonding strength of APTMS, amine‐PDMS linker and bis‐amino silane, over long‐term incubation under cell culture conditions. Membranes were bonded to a featureless PDMS square and completely immersed in cell culture medium over a period of 10 days. Under these conditions, structures treated with either APTMS or amine‐PDMS linker, showed complete delamination after a period of 48 h (**Figure** **4**a). Bis‐amino silane was the top performer over long‐term incubation, forming a permanent bond between PET and PDMS after a 10‐day period of incubation, without observable delamination. When membranes silanized with this method were pulled from PDMS, the PET‐PDMS interface remained strong and the assembly teared at a random position (Figure 4b). This observation is a critical factor as biochips intended for cell culture applications are operated over an extended period, under high humidity and physiological temperatures. Therefore, for all further experimentation PET‐PDMS bonding was performed by silane coupling with bis‐amino silane. Bonding efficacy was further confirmed by monitoring cell loading and growth on Device 2 (Figure 1b), over a period of time. No extravasation to the lower compartment was noticeable during seeding and cells were kept for the entirety of the run within the confines of the cell culture chamber, without invasion of the PET‐PDMS contact zone (Figure 4c). The success of the bonding procedure must rely not only in the ability of the structure to withstand internal pressure, but also on its ability to maintain a stable bond over an extended period, in a humidified atmosphere. Surface passivation with bis‐amino silane was the most reliable surface treatment regarding both aspects considered. The procedure selected combines O_2_ plasma oxidation of the surfaces and silanization of PET materials with bis‐amino silane, for long standing PDMS‐PDMS and PET‐PDMS bonding. Furthermore, we did not observe a correlation between delamination and geometry of the designs. Access portholes, fluid routing channels and cell culture chamber, all remained sealed throughout experimentation, regardless of geometry.

### 2.4 On‐Chip Conditions and PET Treatment do not Affect Cellular Homeostasis

**Figure 5:**
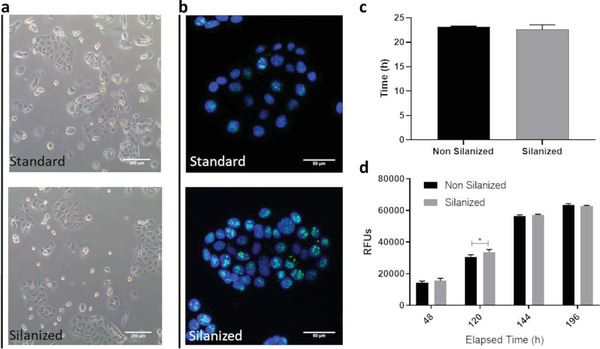
a) Morphological study of phenotype of MKN74 cells growing under standard conditions with cells growing over silanized surfaces (scale bar 200 µm). b) Ki67 staining of the same populations (scale bar 50 µm). c) Population doubling time. d) Comparative study of the metabolic activity of cells growing on a non‐silanized versus silanized surface. All graphical representations display average measurement ± SD (*n* = 6).

Fabrication methods intended for cell culture applications require consideration regarding the biocompatibility of all the materials and chemistries used. To assess these aspects, we used a human stomach adenocarcinoma epithelial cell line MKN74, as a biological model. These cells typically grow a confluent monolayer displaying selective paracellular permeability,^[^ ^33^ ^]^ with fully functional cell‐to‐cell contacts and a typical cobblestone‐like, epithelial geometry (**Figure** **5**a—top plate). Experiments were performed on 24‐well plates, under conditions identical to those found on‐chip. This is important to better benchmark the performance of these cells compared to those growing under conventional cell culture conditions. When applicable, the surface of the 24‐well PET plate was coated with bis‐amino silane. We compared proliferation and doubling time of cells growing over a non‐silanized surface, against cells growing over a silanized surface. Our observations showed no phenotypical differences between both groups (Figure 5a). Cells of both experimental groups were stained 48h post‐seeding, against the proliferation marker Ki67. At this time‐point, both were fully proliferative (Figure 5b). This was further evidenced by the observation that the doubling time was similar for both groups (Figure 5c). Finally, the metabolic activity was measured over a period of 8 days. Our results showed no difference between cells seeded on a silanized or a non‐silanized surface (Figure 5d). Overall, our observations demonstrate that the surface treatment employed to permanently bond the PET membrane to PDMS, does not affect cellular homeostasis, nor does it induce cytotoxicity.

We have established a fabrication procedure that generates stable, long‐term operating devices under physiological conditions. Furthermore, silanization of the intercalating PET membranes does not adversely affect cellular maintenance, validating the procedure for cell culture applications.

### 2.5 Built‐In Microactuator Allows Mechanical Stretching at Physiological Levels and Impacts On‐Chip Cellular Organization and Molecular Signature

**Figure 6:**
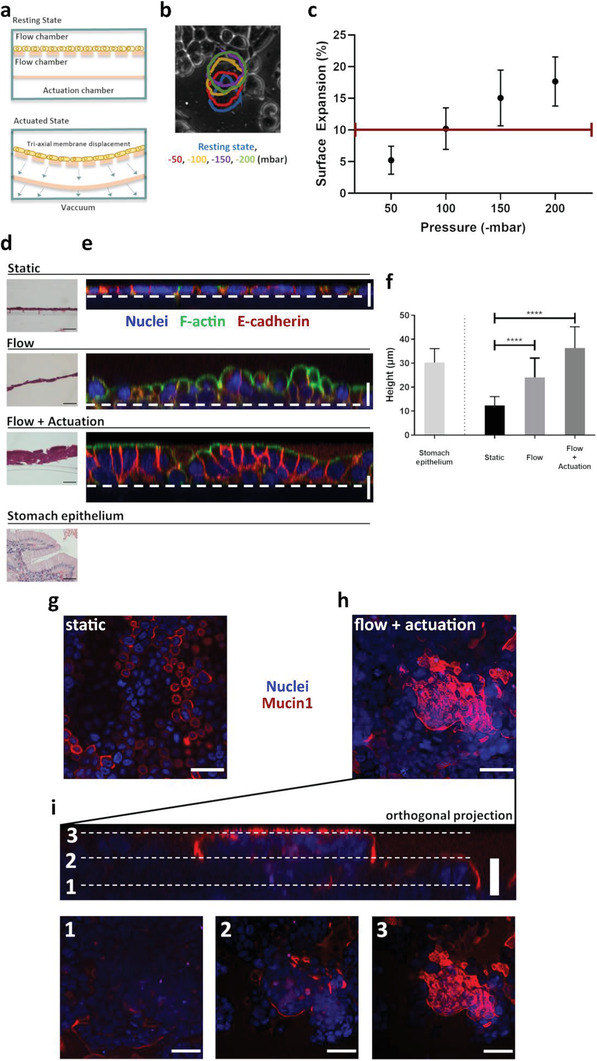
a) Illustration of on‐chip microactuator. b) Vacuum pressure is applied at the actuation chamber, resulting in 3D cell surface expansion. Color‐coded outlines exemplify cellular expansion for a single cell at different actuation pressures. c) Surface expansion as estimated from the linear expansion, at different pressure steps; red line represents average surface tension of in vivo gut cells.^[^ ^34^ ^]^ d,e) Qualitative comparison of epithelium height of MKN74 cells grown under static (transwell) versus dynamic conditions (flow or flow coupled with actuation) d) stained with HE or e) stained for F‐actin (green) and E‐cadherin (red); nuclear material stained with DAPI (blue). The white line highlights the PET membrane position. f) Quantitative study of epithelium height of MKN74 cells grown under static versus dynamic conditions. Quantification was performed using orthogonal projections of z‐stacks for each condition (z‐step: 2 µm), except for normal stomach epithelium, which was quantified from HE stained specimens (*n* = 3). Epithelium height was measured using image analysis software (Fiji). g,h) Mucin‐1 staining (red) of MKN74 cells grown under static or dynamic conditions (flow coupled with actuation), respectively (scale bar: 50 µm). Images represent a maximum intensity projection of a z‐stack, post‐processed using image analysis software (Fiji). Nuclear material stained with DAPI (blue). i) Orthogonal projection of (h). Bottom plates represent the same microscopic field at 3 different z‐heights. All graphical results presented as average measurements ± SD. Scale bar for orthogonal projections correspond to 20 µm and for HE images to 40 µm.

Mechanical stretching in living tissues is rarely of a single plane, linear nature. Hence, the microactuator was designed so that membrane displacement is applied from below the cell culture substrate (Figure 1c). By applying vacuum to the microactuator chamber, the flexible membrane above it is actuated in 3D, which in turn displaces the cell culture substrate above it (**Figure** **6**a). Cell culture substrate stretching was assessed at 0, −50, −100, −150, and −200 mbar. At vacuum pressures below −200 mbar, within our 250 µm high actuator, we observed that the flexible PDMS membranes touched the bottom of the microactuator cavity. As a result, no measurements were recorded below this threshold since the stronger vacuum pressure did not reflect in further stretching of the membrane. In order to quantify the membrane stretching we photographed the porous membrane at resting state and at actuated state and measured the interpore distance, to calculate the linear expansion effected. Surface expansion was then estimated numerically from the values obtained for the linear expansion and, in addition, we visually assessed the perimeter of expanding cells under actuation (Figure 6b). Within the pressure ranges tested we achieved surface expansion values between 5±2% at −50 mbar, up to 18±4% at −200 mbar (Figure 6c). These values are well within the surface expansion experienced by cells within living tissues of some of the most common organ‐on‐a‐chip models, where cell stretching is an important physiological factor, namely gut,^[^ ^34^ ^]^ lung ^[^ ^35^ ^]^ and heart.^[^ ^36^ ^]^ To further investigate the ability of the built‐in microactuator, to induce physiological level alterations to the cell culture within the biochip, we performed a morphological and molecular characterization of a gastric epithelial population growing on‐chip, under dynamic conditions. To emulate peristalsis‐like motion similar to that experienced by gut cells in vivo, the stretching pattern was modelled as a sinusoidal wave of 0,15 Hz frequency, and maximum vacuum pressure of −100 mbar applied to the microactuator. These conditions generated a surface expansion around 10% as shown in Figure 6c and promoted a range of physiological strain similar to that experienced by gut cells in vivo.^[^ ^14^, ^34^, ^37^ ^]^ To emulate the gastric epithelium, we have used the MKN74 cell line. We have previously shown that these cells correctly express and localize adherens‐junctions and tight‐junctions partners when cultured in static conditions,^[^ ^33^ ^]^ which promotes the establishment of a tightly knit epithelial barrier, once confluency is reached. Furthermore, our previous results show that once confluency is reached, transepithelial resistance rapidly increases, reaching a plateau above 150 Ω.cm^2^, at day 5 post‐seeding and lasting at least 11 days.^[^ ^33^ ^]^ Taking this in consideration, we have devised an experimental setup to assess the influence of dynamic conditions on a fully developed and selective epithelium. A confluent MKN74 population was cultured to confluency, taking on average 3 days, followed by 3 days under culture media flow alone or, in addition, with fluid flow and peristaltic actuation. This timescale provides an experimental window where the newly formed epithelium is properly packed and exhibiting selective permeability, similar to the normal gastric mucosa. A control population was also monitored, by growing for the same period of time MKN74 cells in transwell inserts under static conditions. We have observed that irrespective of the condition tested, cells reached confluency at around day 3 in culture when seeded at a density of 3,0×10^5 ^cells/mL. Cells formed a compact layer which was undistinguishable on a phase contrast microscope (data not shown). Interestingly, once mechanical stretching was initiated, a marked difference was observable. MKN74 cells under mechanical actuation, seemed to develop a much thicker epithelial layer (Figure 6d). To further understand the parameters being effected by the mechanical stretching over the cell culture substrate, we stained cells with an antibody against the adherens‐junction protein, E‐cadherin and assessed distribution of the F‐actin cytoskeleton. Here, we observed that cells grown under static conditions displayed a flattened, undifferentiated appearance, identical to a squamous epithelium (Figure 6e—top plate). In contrast, cells under dynamic conditions, exhibit a markedly different phenotype. Flow alone, induced an increase in cell height and the formation of globular‐like structures (Figure 6e—middle plate). The effect was even more striking on those cells under flow and peristaltic actuation. Furthermore, cells display an elongated shape, resembling a polarized state, with F‐actin observable across the whole membrane, while E‐cadherin was limited to the basolateral region of the cells. Interestingly, nuclear staining showed that nuclei are elongated and localized to the basal side of actuated cells, further evidencing differentiation traits that are not commonly found in gastric cells under static conditions (Figure 6e—bottom plate). We then compared average size of the engineered gastric epithelia. Our observations suggest that on‐chip dynamic conditions are capable of eliciting a response at the cellular level, which is initiated with flow alone. Average epithelium height increased 2‐fold in cells under media flow alone, when compared to static conditions. The increase was even more striking when considering cells under flow coupled with mechanical actuation, with an almost 3‐fold increase when compared to the epithelium height of cells under static conditions. Interestingly, the average height of the epithelium generated from actuated cells (approximately 36 µm), is similar to that observed in normal stomach epithelium (approximately 30 µm) (Figure 6f). Despite these findings, our observations suggest that the actuated epithelium resembles a pseudostratified columnar epithelium, rather than a simple columnar epithelium as found in normal gastric mucosa (Figure 6d—bottom plate). Finally, we studied expression of Mucin‐1. In normal stomach mucosa, this protein is located at the apical surface of epithelial cells.^[^ ^38^ ^]^ For cells under static conditions, expression of Mucin‐1 was limited to single dispersed cells (Figure 6g). The mechanically actuated epithelium however, displayed zones of globular growth, where Mucin‐1 was exclusively expressed at the apical surface (Figure 6h), closely resembling the expression of Mucin‐1 in normal gastric mucosa. This is also in accordance with what has been previously demonstrated for 3D MKN74, gastric spheroids.^[^ ^39^, ^40^ ^]^

**Figure 7:**
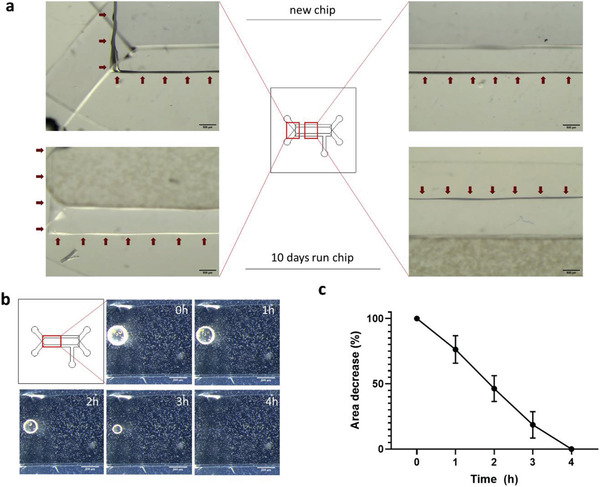
a) Assessment of microactuator delamination over a period of 10 days. Top plates show a new chip prior to use. Red arrows point to the limits of the microactuator. Part of the flow channel network can be seen out of focus. The bottom plates show the detail of a chip that has been run for 10 days. No delamination of the microactuator was observed (red arrows). Fluidic channel with seeded MKN74 cells can be seen out of focus, above the microactuator (scale bars: 500 µm). b) In‐line air bubble degassing by application of a constant −50 mbar vacuum pressure at the microactuator over a period of time. c) Area of each bubble was measured using image analysis software (Fiji) and plotted as % of area decrease over‐time. Results presented as average measurements ± SD.

Overall, our data demonstrates that the proposed fabrication method can effectively be used to engineer biochips with fully integrated mechanical actuators without adding complexity to the fabrication procedure. The developed system reproduces mechanical strain at near physiological levels, suitable for a variety of organ‐on‐a‐chip models, when substrate actuation is required. Furthermore, using a gastric epithelial model, we have demonstrated that the engineered microactuator is capable of eliciting a physiological response at the molecular level, by inducing differentiation and polarization traits, characteristic of the normal gastric mucosa. Finally, our observations show that biochips fabricated with this method can be reliably used for long term experimentation, as no delamination of the microactuator components was observed over a period of 10 days under use (**Figure** **7**a).

### 2.6 Built‐In Microactuator Allows In‐Line Degassing of Air Bubbles

A common complication observed during microfluidic operation is the formation of air bubbles.^[^ ^41^ ^]^ Air bubbles can disrupt flow and the creation of an air‐liquid interface may compromise cellular homeostasis and promote cellular death. Biochips that need to be operated at 37°C are particularly susceptible to this problem, as the higher temperature leads to decreased gas solubility.^[^ ^41^ ^]^ Under these circumstances, the volume of the air bubble will gradually expand over time. Taking advantage of the fact that the microactuator was entirely fabricated in PDMS, which is permeable to gas exchange, we tested its potential operation as an integrated bubble degasser using Device 3 (Figure 1c). As such, we monitored its ability to eliminate air bubbles from the flow path over time. We injected air in the main flow line and pushed the air bubbles up to the main culture chamber, at which point the chip was placed at 37°C inside the cell culture incubator and a constant vacuum pressure of −50 mbar was applied, while monitoring air bubble area, every hour. Our results show that the vacuum actuator was able to effectively eliminate air bubbles over‐time from the main culture chamber (Figure 7b). We observed that a −50 mbar constant vacuum pressure could effectively eliminate a bubble with an average area of 250.5 mm^2^ over a period of 4 h (Figure 7c). This is an interesting observation, as it solves a common problem with standard microfluidic systems, without further complicating the architecture of the chip or increasing fabrication time. Taking in consideration these observations, we added a degassing step to our chip priming procedure. After sterilization and surface coating, but before cell seeding, the chip was allowed to equilibrate overnight at 37°C, while applying a constant vacuum pressure of −50 mbar. As the system was pressurized and fully contained throughout operation, this procedure was sufficient to prevent air bubble formation during experimental procedures.

## 3 Concluding Remarks

Advances integrating microengineering and tissue engineering enabled the creation of organ‐on‐a‐chip platforms, which aim is to recapitulate the micro‐physiological and functional characteristics of human tissues. In particular, organ‐on‐a‐chip devices excel in their ability to modulate the cellular response to mechanical cues, one aspect that is often underrepresented in conventional 2D culture models.^[^ ^42^ ^]^ Modelling the complex tissue response at a microscale, requires devices of complex architecture and functionality. Soft lithography is ideally suited for the fabrication of organ‐on‐a‐chip platforms, given the biocompatibility, optical transparency and, importantly, the elastomeric nature of PDMS that enables the development of mechanical actuators. However, soft lithography is a time‐consuming procedure and requires specialized equipment and training.

In this work, we described a fully modular, rapid prototyping, alternative method for the fabrication of complex multilayered microfluidic devices with integrated microactuators. Our approach reduces the complexity, number of steps, time and cost required to fabricate a functional microfluidic chip. We based our approach on xurography, a technique based on the removal of material from thin film plates using a cutting plotter.^[^ ^20^ ^]^ Here we have explored a similar method to that described by Cosson and colleagues, by using a cutting plotter to directly machine thin laminated PDMS sheets.^[^ ^21^ ^]^ In this work however, we have used commercially available thin PDMS laminates, thus eliminating the lengthy process of producing thin PDMS sheets, namely mixing/degassing PDMS, spin coating each individual PMDS sheet and fully curing them, reducing overall fabrication time. Structures fabricated were shown to have a similar resolution to those obtained by Cosson and colleagues. Good precision can be achieved in the micrometric range, with 200 µm structures at the lower end of resolution attainable. The described approach obviates the most time‐consuming portion of the soft lithographic method, i.e., the fabrication of molds and the casting of individual parts. A full device can be plotted within seconds, from a strip of PDMS laminate. Despite this, each plotted PDMS layer and corresponding intercalating membranes, must still be assembled manually, using the same procedures employed in conventional soft lithography.

The full modularity of our fabrication method constitutes also a valuable characteristic for the early prototyping stages, as each aspect of the chip, i.e., chip robustness, cell culture maintenance and mechanical stretching, can be tested independently before new layers are added. Alterations to the design can thus be quickly implemented and tested. Importantly, we have demonstrated that our method permits the facile integration of microactuators without further complicating the design or fabrication process. Our prototype was able to stably sustain the effects of strain on the cell culture substrate at near physiological levels. Furthermore, we have demonstrated using a gastric model that microactuators fabricated with the proposed technique, are capable of eliciting a cellular and molecular response, inducing physiological level alterations to the cells growing within the chip. Devices were shown to be pressure resistant and performed well under cell culture conditions over a long period, as no delamination or leakage was observed. A combination of O_2_ plasma exposure and chemical modification of membranes with a commonly used silane, namely bis‐amino silane,^[^ ^31^ ^]^ proved to be an effective process to produce leak‐tight devices, without affecting viability or cellular homeostasis. Furthermore, the versatility of the method was demonstrated by the successful adaptation of the microactuators as in‐line degassers, eliminating air bubble formation during operation, which is one of the major challenges during biochip handling.

The described method can significantly contribute to the early stages of organ‐on‐a‐chip development by reducing 3 of the main burdens facing a microfabrication laboratory, namely production time, cost and space requirement. It presents a fully modular, low cost and fast alternative to soft lithography, while retaining all the advantages of PDMS‐based cell culture devices. Notably, the entire procedure is accomplished with portable benchtop equipment, reducing greatly the benchtop footprint. We are currently working on an advanced model of the stomach mucosa, using the technology described here. Our aim is to explore in a more physiologically relevant context, our previously described work of a 3D gastric mucosa model.^[^ ^33^, ^43^, ^44^ ^]^

## 4 Experimental Section

##### Preparation of Chip‐Related Materials

Prior to assembly, each plate design, was cut from PDMS foil (915 mm wide, MVQ Silicones), using a desktop cutter (model CAMM‐1 GS‐24, Roland DG). The fluidic plates were cut from 500 µm thick foil, whereas the plates forming the pneumatic portion of the device were cut from 250 µm thick foil. Briefly, a section of PDMS foil of about 3×20 cm, was manually cut with scissors and one side of the protective backing plastic was removed. Finally, the laminate sheet was fed to the cutting plotter and the design transferred from the CAD software to a dedicated software, Roland Cut Studio (Roland DG). Cutting was performed with a carbide cemented blade (model ZEC‐U5032, Roland DG). Blade pressure was set at 10 gram‐force and speed at 20 cm.s^–1^. After the machining process, the offcut material was removed with tweezers, to reveal the hollow fluidic and pneumatic features (Figure 1d).

The polyethylene terephthalate (PET) membrane (thickness 16 µm, pore size 8 µm, pore density 6e^4^ cm^2^, it4ip), was cut manually from Ø25 mm discs, immersed in isopropanol (IPA), cleaned by ultrasound (5 min), dried with compressed air and stored in a dust‐free container.

The top layer of each chip was fabricated by gravity casting PDMS on a circular petri dish. The PDMS base was mixed thoroughly with curing agent on a weight ratio of PDMS base to curing agent 10:1 (Sylgard 184, Dow Corning). The heavily aerated pre‐polymer mix was degassed by centrifugation (5 min at 3166 g), carefully poured onto 90 mm petri dish and cured at 70°C for 1 h. The resulting PDMS plate was then cut in 26 mm square sections to match the machined PDMS layers.

Glass microscope slides were thoroughly cleaned by sequential incubation (5 min by ultrasound) in 2% (v/v) Helmanex III solution in double distilled water (Helma Analytics), followed by acetone and a final rinse in double distilled water. Clean slides were air dried with compressed air and stored in a dust‐free container.

##### Microfluidic Device Design and Assembly

To characterize the fabrication method, 3 microdevices were designed (Figure 1). Device 1 (Figure 1a) is a 5‐layered fluidic system, with two fluidic linear channels measuring 12 mm in length, arrayed in a cross format and separated by a PET perforated membrane. Device 2 (Figure 1b) is built from 5 superimposed layers. It is composed by 3 alternating layers of 500 µm PDMS laminate foil and a cell culture treated PET membrane (Figure 1b). Fluidic features are rectangularly shaped, 2 mm (width) x 10 mm (length) x 0.5 mm (height), for a total cell culture area of 0.2 cm^2^ and a total volume of 0.01 cm^3^ (approximately 10 µL). Access to the cell culture area is made through an angular feature connecting to an access porthole of 2 mm in diameter. Fluid flow is applied unidirectionally, serving one porthole as an inlet and the other as an outlet for liquid ejection. Design for Device 3 (Figure 1c

) was adapted from Device 2, by adding a lower portion, containing the pneumatic actuation system. The actuator is a two‐piece assembly comprised of a 0.25 mm (height) flexible PDMS membrane and an actuation chamber with 35 mm (width) x 11 mm (length).

Assembly was performed from top to bottom. PDMS and glass structures were bonded permanently by exposure to O_2_ plasma (1 min, 1 bar; Zepto Plasma Laboratory Unit, Diener) and bringing them into immediate conformal contact. This created a strong and irreversible bond between both layers.^[^ ^26^, ^45^ ^]^ The microchannel outline was used as a guide to punch the inlet and outlet access channels through the top cast‐on PDMS layer, followed by a thorough wash with IPA. Porous membranes were silanized as described below and sandwiched between two O_2_ plasma exposed PDMS plates. Alignment of the fluidic and microactuator features was performed under a stereomicroscope (model SZX10, Olympus). When applicable, the microactuator was sealed below the fluidic assembly. Care was taken to ensure the correct alignment of the flow features with the pneumatic chamber. The process was finalized by sealing the chip assembly against a microscope glass slide.

##### Priming and Operation

Fluid flow and pneumatic actuation were managed with a piezoelectric pressure controller (model OB1 Mk3, Elveflow). The equipment was tethered to a dedicated software (ESI, Microfluidic Software and SDK, Elveflow) and coupled to a flow sensor that allows control over the flow rates applied, up to a maximum of 80 µL/min. Mechanical actuation was achieved by application of vacuum pressure to the microactuator via poly(ether‐ether‐ketone) (PEEK) tubing (Idex).

Prior to operation, the microfluidic device was rinsed with ethanol 70% (v/v) and exposed to one UV cycle (20 min), after which, ethanol 70% was flushed through the fluidic features (15 min), followed by washing with phosphate buffered saline (PBS; 15min) solution. To promote cellular adhesion, the surface of the fluidic features was perfused with a fibronectin solution (50 µg/mL, Sigma) and incubated at room temperature for 2 h. Before connecting the biochip, the perfusion system was sterilized by flushing ethanol 70% (15 min), followed by rinsing with PBS (15 min). The perfusion system was then filled with cell culture medium and afterward the microdevice was connected in‐line and allowed to equilibrate overnight.

##### Cell Culture

The human derived epithelial gastric cancer cell line, MKN74, was purchased from the Japanese Collection of Research Bioresources cell bank. Samples were stored in liquid nitrogen and tested negative for the presence of mycoplasma when thawed, and every month. Cells were routinely maintained in Roswell Park Memorial Institute (RPMI) 1640 medium with glutamax (Gibco), supplemented with 10% (v/v) fetal bovine serum (FBS, Biowest) and 1% (v/v) penicillin/streptomycin (pen/strep; Biowest) at 37°C, 5% CO_2_ and in a high humidity atmosphere. Cells were reseeded every 3 days, or when confluency reached around 80%.

On‐chip, cells were grown in CO_2_‐independent cell culture media (Gibco), supplemented with 4 mM of L‐Glutamine (Gibco), 10% (v/v) FBS (Biowest) and 1% (v/v) pen/strep (Biowest). Prior to on‐chip seeding cells were allowed to stabilize for 48h in CO_2_ independent cell culture media. Cells were then detached by trypsinization and resuspended to a density of ca. 3.0×10^5 ^cells/mL. The resulting suspension was loaded on the upper fluidic channel via a secondary port operated by a 4‐way PEEK valve (Idex). Cells were allowed to sediment and attach for a period of 30 min, after which cell spread and density on the membrane was assessed under a phase contrast microscope. On‐chip cells were cultured at 37°C, in normal, high humidity atmosphere (without CO_2_ buffering). Cell culture media was perfused at 0.5 µL/min for the duration of the experimental work.

##### Actuation Protocol

Cells were seeded on‐chip as detailed above and allowed to reach confluency. Mechanical stretching of the cell culture substrate was then effected by application of cyclic vacuum to the microactuator. In order to emulate peristalsis experienced by gut cells in vivo, the stretching pattern was modelled as a sinusoidal wave of 0.15 Hz frequency^[^ ^14^ ^]^ and maximum vacuum pressure of −100 mbar.

##### Evaluation of Machining Precision

To assess the precision of the cutting plotter, CAD design dimensions were compared with those of machined pieces. For each condition tested, structures (*n* = 4) were photographed under a stereomicroscope (model SZX10, Olympus) coupled with a camera (model EP50, Olympus) and length measurements were performed using image analysis software (Fiji).^[^ ^46^ ^]^ Three key geometries (circular, angular and linear features) were measured in quadruplicate and results graphed as plotted dimensions against theoretical dimensions. Deviation to CAD size was plotted as coefficient of variation (%).

##### PET‐PDMS Bonding

Permanent bonding between PET membranes and PDMS laminates, requires surface treatment. A process of PDMS stamping was tested, as well as 4 different silanization protocols.

PDMS stamping was adapted from a previously described work.^[^ ^27^ ^]^ Briefly, a batch of uncured PDMS pre‐polymer was mixed in a ratio of PDMS base to curing agent of 10:1 and the borders of the PET membrane were immersed in this mixture. The surface of the PDMS laminate was also thinly coated with the uncured PDMS and both materials were pressed together and cured at 120°C for 1 h.

Silanization of (3‐aminopropyl)trimethoxysilane (APTMS) and (3‐aminopropyl)triethoxysilane (APTES) was applied by vapor deposition. Briefly, clean membranes were exposed to a silane saturated atmosphere for 15 min, followed by a baking at 70°C for 1 h. Membranes were then rinsed with IPA and both the membrane and the PDMS surface were exposed to O_2_ plasma (1 min) and placed in contact. Pressure was applied overnight at room temperature.

Silane treatment with poly[dimethylsiloxane‐co‐(3‐aminopropyl)methylsiloxane] (amine‐PDMS linker) was performed by direct surface contact. The protocol was adapted from Wu J. et al.^[^ ^32^ ^]^ Briefly, the PET membranes were covered with a droplet of amine‐PDMS linker and exposed for 20 min to the amine‐PDMS linker. To remove excess silane, membranes were immersed in IPA and sonicated for 1 min. PDMS and membranes were exposed to O_2_ plasma (1 min) pressed together and baked at 80°C for 1 h. Surface passivation with bis[3‐(trimethoxysilyl)propyl]amine (bis‐amino silane) was performed by securing the membranes vertically onto a PDMS support and exposing them to O_2_ plasma (1 min). This procedure was followed by immediate incubation in 2% (v/v) bis‐amino silane, 1% (v/v) double distilled water in IPA solution (20 min at 80°C). Membranes were washed thoroughly with IPA and cured for 30 min at 70°C. Prior to bonding, membranes were wetted for 30 min in ethanol 70% (v/v) and pressed against O_2_ plasma exposed (1 min) PDMS laminates.

To test resistance to delamination of the PET‐PDMS bond, Device 1 was used (Figures 1a and 3a). Both perfusion channels were filled with double distilled water, dyed with red food coloring and 3 of the outlets were plugged with 20Ga metal studs (Figure 3a—right plate). The remaining inlet was plugged to the pressure controller via polyethylene tubing (Instech). Pressure was applied in 100 mbar increments, up to 1000 mbar. The device was kept at each pressure step for 30 min after which the PET‐PDMS interface was photographed under an inverted microscope (model IX71, Olympus).

Delamination under cell culture conditions was assessed by bonding a silanized PET membrane onto a featureless PDMS laminate and immersing the assembly in cell culture media. This assembly was then placed inside a cell culture incubator for 10 consecutive days, after which it was removed from the petri dish and the membrane was forcefully pulled from the PDMS with tweezers.

##### Metabolic Activity and Proliferation Rate

Metabolic activity was assessed by the resazurin assay. Approximately 100 cells/well were seeded in a 24‐well plate and allowed to adhere for 24 h. Metabolic activity was assessed every 48 h for 15 days. For the measurement of metabolic activity, a stock solution of 0.1 mg/mL resazurin (Sigma) was diluted to 20% (v/v, resazurin/cell culture media). Culture media was replaced with 500 µL of this solution and incubated at 37°C for 2 h. 200 µL of the resulting supernatant were transferred to an opaque 96‐well plate with clear bottom (Greiner) and the fluorescence signal was measured (Ex 530 nm/Em 590 nm) in a fluorimeter (model Sinergy MX HM550, Biotek Instruments). Metabolic activity was expressed as average relative fluorescence units (RFUs) ± standard deviation (SD) (*n* = 6).

The population doubling time was estimated from the metabolic activity results registered between 0 and 48 h, at which point cells were non‐confluent and growing at an optimal exponential rate.

##### Immunocytochemistry (ICC)

ICC against Ki67, a marker of proliferation, was performed on a non‐confluent population, 48 h post‐seeding. ICC against F‐actin, E‐cadherin and Mucin‐1, was performed on populations grown in transwell systems under static conditions or on populations grown on‐chip, under dynamic conditions. Briefly, cells were washed in PBS with 0.05 mg/mL of CaCl_2_ (3×5 min), fixed with 4% (v/v) paraformaldehyde suspended in PBS (20 min at room temperature, Electron Microscopy Sciences). Fixation was followed by further washing with PBS (3×5 min), followed by incubation in 50 mM NH_4_Cl (10 min) and permeabilization with 0.2% (v/v) triton‐X100 (5 min). Cells were rinsed with PBS (3×5 min) and blocked in 5% (v/v) bovine serum albumin (BSA) in PBS (30 min). Primary antibody incubation was performed with rabbit anti‐Ki67 (dilution 1:100 in 5% (v/v) BSA; Abcam), rabbit anti‐E‐cadherin (24E10, dilution 1:100 in 5% (v/v) BSA, Cell Signaling), mouse anti‐Mucin‐1 (dilution 1:6, kindly gifted by Dr. Celso Reis ‐ from i3S/Ipatimup, Portugal), overnight at 4°C. Secondary reaction was done with anti‐rabbit alexa fluor 594 antibody (dilution 1:500; Thermo Scientific) or anti‐mouse alexa fluor 594 antibody (dilution 1:500; Thermo Scientific), for 2 h at room temperature. When applicable, secondary antibody was co‐incubated with phalloidin (Biolegend) to stain the actin cytoskeleton. After rinsing with PBS (3×5 min), cellular preparations were mounted in vectashield containing 2‐(4‐amidinophenyl)‐1H‐indole‐6‐carboxamidine (DAPI), for nuclei staining. Image acquisition was done in a confocal microscope (model SP5, Leica Microsystems).

For hematoxylin and eosin (HE) staining of membrane bound epithelial cells, sample membranes were removed from their supporting structure and fixed as described above. Samples were then embedded in optical cutting temperature compound, frozen, and then 3 µm sections were obtained using a cryostat. The sections were washed in double distilled water, stained 3 min in Gill's hematoxylin, incubated 6 min in running water, dehydrated, stained 1 min in eosin Y, cleared and mounted in entellan. Images were acquired with a multihead optical microscope (Zeiss).

##### Epithelium Characterization

The in vitro epithelium was fixed and F‐actin was stained with phalloidin and co‐incubated with an antibody against E‐cadherin as described above. Z‐stacked fluorescence images were acquired with a 2 µm z‐step value, on a confocal microscope (model SP5, Leica Microsystems). Z‐stacks were post‐processed and epithelium height measured using image analysis software (Fiji). Normal stomach mucosa height was measured from HE stained specimens. Non‐cancerous/normal gastric mucosa samples were obtained and used with consent from the Tissue and Tumour Biobank of CHSJ/Ipatimup.^[^ ^47^ ^]^

##### Surface Expansion

To assess surface expansion, a biochip (Device 3; Figure 1c) was filled with cell culture media and connected to the piezoelectric pressure controller using PEEK tubing. The magnitude of negative pressure applied was managed by the pressure controller, by creating a vacuum generated pull on the flexible PDMS membrane that, in turn, displaced the perforated cell culture membrane above it. By applying mechanical distension from below, the membrane is actuated three dimensionally by pulling and releasing in the x, y and z planes. Cell culture substrate stretching, was assessed at 0, −50, −100, −150, and −200 mbar. Still images were taken with an inverted microscope (model IX71, Olympus), at rest and actuated state and the average interpore distance (distance between two adjacent pores) in both states was measured using image analysis software (Fiji). Linear expansion was calculated as the amount of linear stretch sustained by the substrate according to the following equation:

(1) $$\varepsilon_{\mathrm{lin}}=\left(L_{f}-L_{0}\right)/L_{0}$$

where L_0_ and L_f_ are the interpore length before and after actuation, respectively. Surface expansion was estimated from the linear expansion using the following formula:

(2) $$\varepsilon_{\mathrm{SA}}={\left(\varepsilon_{\mathrm{lin}}+1\right)}^{2}-1$$

*Ɛ*_SA_ is the surface area expansion and *Ɛ*_lin_ is the linear expansion. The results were plotted as percentage of *Ɛ*_SA_ ± SD relative to the rest state.

##### In‐Line Degassing Protocol

After priming, the chip was filled with PBS solution and allowed to equilibrate at 37°C for at least 2 h. Air bubbles were injected through the system and pushed until they were inside the cell culture chamber. A constant vacuum pressure of −50mbar was applied on the microactuator and the air bubbles were photographed every hour under a stereomicroscope (Olympus SZX10) coupled with a camera (Olympus EP50), to monitor air bubble area variation.

##### Statistical Analysis

Data was compiled and analyzed using the software Graphpad Prism (Graphpad Software). Data plotted in graphical format is presented as mean value ± SD, except deviation to CAD theoretical dimension, which was represented as coefficient of variation (%). Experimental determination of resolution limits tested with an *n* = 4. Cell culture doubling time and metabolic rates (*n* = 6) were compared using a two‐way ANOVA statistical test. The latter were analyzed in conjunction with a Sidak's post hoc multiple comparison test. Epithelium height measurements (*n* = 3) were compared using an unpaired t‐test. Statistical significance was defined as p<0.05 (*), p<0.01 (**), p< 0.0001 (****).

## Conflict of Interest

The authors declare no conflict of interest.
